# Cell cycle time series gene expression data encoded as cyclic attractors in Hopfield systems

**DOI:** 10.1371/journal.pcbi.1005849

**Published:** 2017-11-17

**Authors:** Anthony Szedlak, Spencer Sims, Nicholas Smith, Giovanni Paternostro, Carlo Piermarocchi

**Affiliations:** 1 Department of Physics and Astronomy, Michigan State University, East Lansing, Michigan, United States of America; 2 Salgomed Inc., Del Mar, California, United States of America; 3 Sanford Burnham Prebys Medical Discovery Institute, La Jolla, California, United States of America; University of Chicago, UNITED STATES

## Abstract

Modern time series gene expression and other omics data sets have enabled unprecedented resolution of the dynamics of cellular processes such as cell cycle and response to pharmaceutical compounds. In anticipation of the proliferation of time series data sets in the near future, we use the Hopfield model, a recurrent neural network based on spin glasses, to model the dynamics of cell cycle in HeLa (human cervical cancer) and *S. cerevisiae* cells. We study some of the rich dynamical properties of these cyclic Hopfield systems, including the ability of populations of simulated cells to recreate experimental expression data and the effects of noise on the dynamics. Next, we use a genetic algorithm to identify sets of genes which, when selectively inhibited by local external fields representing gene silencing compounds such as kinase inhibitors, disrupt the encoded cell cycle. We find, for example, that inhibiting the set of four kinases *AURKB*, *NEK1*, *TTK*, and *WEE1* causes simulated HeLa cells to accumulate in the M phase. Finally, we suggest possible improvements and extensions to our model.

## Author summary

Cell cycle—the process in which a parent cell replicates its DNA and divides into two daughter cells—is an upregulated process in many forms of cancer. Identifying gene inhibition targets to regulate cell cycle is important to the development of effective therapies. Although modern high throughput techniques offer unprecedented resolution of the molecular details of biological processes like cell cycle, analyzing the vast quantities of the resulting experimental data and extracting actionable information remains a formidable task. Here, we create a dynamical model of the process of cell cycle using the Hopfield model (a type of recurrent neural network) and gene expression data from human cervical cancer cells and yeast cells. We find that the model recreates the oscillations observed in experimental data. Tuning the level of noise (representing the inherent randomness in gene expression and regulation) to the “edge of chaos” is crucial for the proper behavior of the system. We then use this model to identify potential gene targets for disrupting the process of cell cycle. This method could be applied to other time series data sets and used to predict the effects of untested targeted perturbations.

## Introduction

Originally proposed by Conrad Waddington in the 1950s [1] and Stuart Kauffman in the 1970s [2], analysis of biological processes such as cellular differentiation and cancer development using attractor models—dynamical systems whose configurations tend to evolve toward particular sets of states—has gained significant traction over the past decade [3–12]. One such attractor model, the Hopfield model [13], is a type of recurrent artificial neural network based on spin glasses. It was designed with the ability to recall a host of memorized patterns from noisy or partial input information by mapping data directly to attractor states. A great deal of analytical and numerical work has been devoted to understanding the statistical properties of the Hopfield model, including its storage capacity [14], correlated patterns [15], spurious attractors [16], asymmetric connections [17], embedded cycles [18], and complex transition landscapes [19]. Due to its prescriptive, data-driven design, the Hopfield model has been applied in a variety of fields including image recognition [20, 21] and the clustering of gene expression data [22]. It has also been used to directly model the dynamics of cellular differentiation and stem cell reprogramming [23, 24], targeted inhibition of genes in cancer gene regulatory networks [25], and cell cycle across various stages of cellular differentiation [26].

Techniques for measuring large scale omics data, particularly transcriptomic data from microarrays and RNA sequencing (RNA-seq), have become standard, indispensable tools for observing the states of complex biological systems [27–29]. However, analysis of the sheer variety and vast quantities of data these techniques produce requires the development of new mathematical tools. Inference and topological analysis of gene regulatory networks has garnered much attention as a method for distilling meaningful information from large datasets [30–36], but simply analyzing the topology of static networks without a signaling rule (e.g. differential equations, digital logic gates, or discrete maps) fails to capture the nonlinear dynamics crucial to cellular behavior. The non-equilibrium nature of life implies that it can only be truly understood at the dynamical level, necessitating the development of new methods for analyzing time series data. As experimental methods continue to improve, more and more high-resolution time series omics and even multi-omics [37] data sets will inevitably become available. Here, we demonstrate that time series omics data (in this case, transcriptomic data) representing cyclic biological processes can be encoded in Hopfield systems, providing a new model for analyzing the dynamics of, and exploring effects of perturbations to, such systems.

The dynamics of cell cycle (CC)—the process in which a parent cell replicates its DNA and divides into two daughter cells—is both scientifically interesting and therapeutically important, and has been modeled extensively using differential equations, Boolean models, and discrete maps [38–55]. Even relatively simple simulated systems such as an isolated, positively self-regulating gene subject to noise can exhibit rich dynamical behavior [56]; but like many biological processes, the proper functioning of CC requires the decentralized, coordinated action of hundreds of genes. CC thus provides researchers with a convenient case study of self-organization in a noisy environment. CC is also an upregulated process in many forms of cancer [57–60], and control of CC using pharmaceutical compounds such as kinase inhibitors is a critical goal in cancer research. The combinatorics of selectively inhibiting sets of genes makes exhaustive experimental searches difficult or impractical [61]. However, network-based mathematical models such as the one presented here enable researchers to examine the effects of perturbations to complex systems [62, 63] by testing potential inhibition targets *in silico*. The efficacy of these predictions can then be experimentally validated or invalidated, providing new information and insights to further refine models.

The remainder of this article is structured as follows. In the Models subsection of the Results section we first discuss how periodic genes were identified in the time series gene expression data sets, and how Boolean attractors were extracted from the continuous data (explained in greater detail in the Methods section). We then introduce the Hopfield model and discuss the specific form of the coupling matrix used in this application. We discuss how to interpret the results of Hopfield simulations in the context of gene expression and cells. We also explain the objective function used by the genetic algorithm to identify potential inhibition targets, designed with the intention of disrupting CC. In the Dynamical behavior subsection, we show that this model qualitatively recreates experimental gene expression data, and we demonstrate and analyze some dynamical properties of the delayed Hopfield model, including the role played by noise. We include supplementary videos to emphasize the dynamical nature of this model. Optimal control fields for both unconstrained searches (in which any gene may be inhibited) and searches constrained to kinases are discussed. Finally, we recap our results and suggest possible improvements and generalizations to our methods in the Discussion section.

## Results

### Model

#### Periodic gene selection

**Fig 1:**
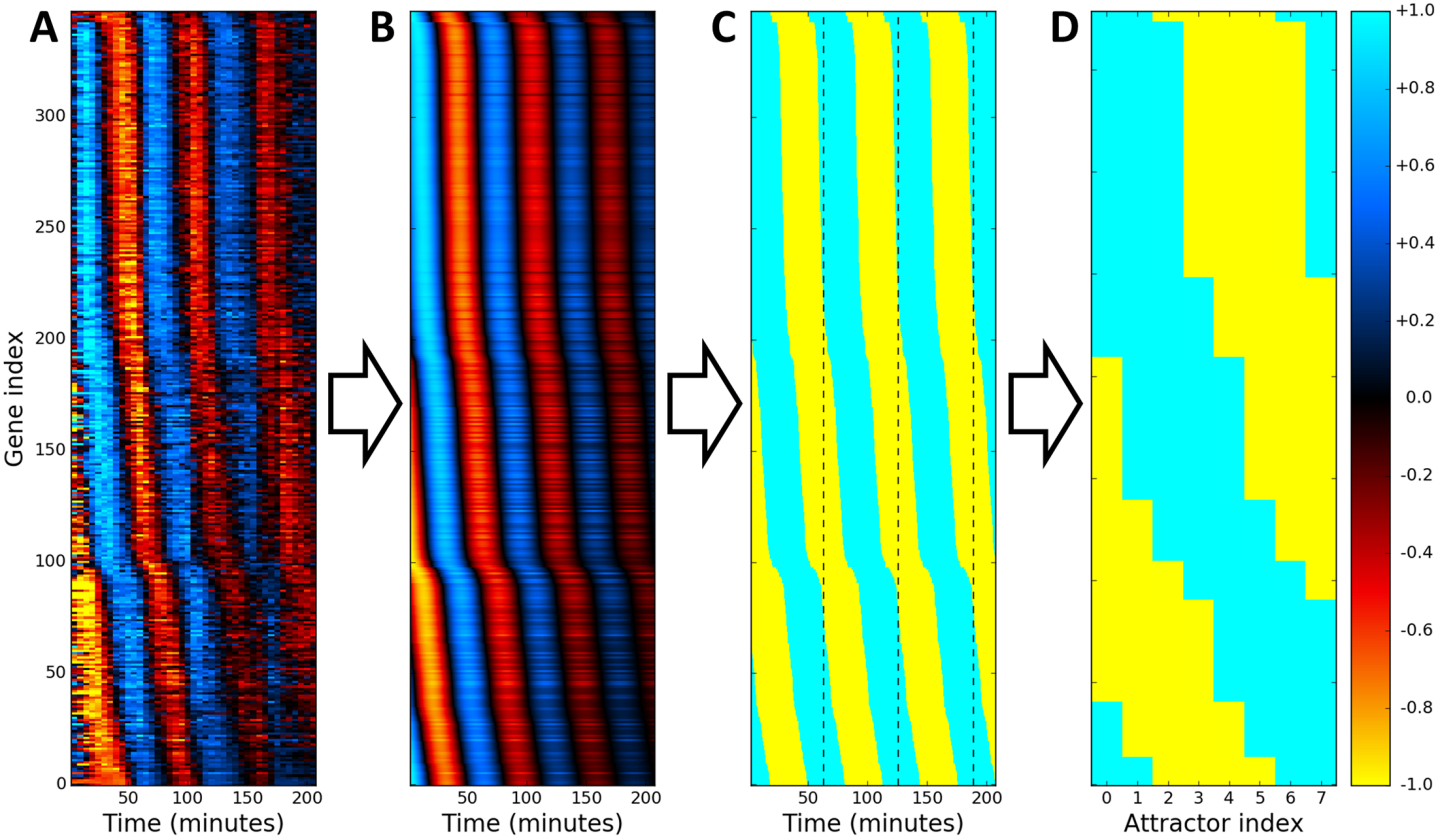
Obtaining attractors from expression data. (A) Heat map of the expression of all detected periodic genes from [64] sorted by their fitted phases. (B) Fitted gene expression. (C) Boolean form of fitted expression, separated into periods by dashed black lines. (D) Final set of *p* = 8 attractors taken from one period.

Microarray and RNA-seq time series data sets were obtained from Eser et al. (*S. cerevisiae*) [64] and Dominguez et al. (HeLa, human cervical cancer) [65]. For consistency and due to its higher resolution, the *S. cerevisiae* data set was chosen to produce all images and movies in this article, but both data sets were analyzed. In order to encode these CC data sets into the Hopfield model, periodic genes needed to be identified, their frequencies and phases computed, and their expression converted from continuous to Boolean form. As detailed in the Methods section, decaying sinusoids were fitted to the trajectory of each gene *i*, and genes with sufficiently high quality fits were kept. This resulted in 379 periodic genes in *S. cerevisiae* and 722 periodic genes in HeLa cells. Fig 1A shows a heat map of the expression of all periodic genes detected in the Eser data set sorted by their fitted phases, and Fig 1B shows the same genes with the fitted expression curves. These fitted curves were converted from continuous values *x*_*i*_(*t*) ≥ 0 to Boolean values *ξ*_*i*_(*t*) = ±1 (over/underexpressed) as shown in Fig 1C. Finally, one CC period was divided into eight uniformly spaced states $\left\{\xi_{i}^{\mu }\right\}=\left\{\xi_{i}^{0},\xi_{i}^{1},\ldots ,\xi_{i}^{7}\right\}$ with $\xi_{i}^{\mu }=\pm 1$. These states, shown in Fig 1D, were used as the eight attractor patterns in the Hopfield model. *p* = 8 was chosen because the embedded cycle lost stability for *p* > 8 due to the finite capacity of the Hopfield model.

Note that this work is based on the simplifying assumption that cell cycle genes exhibit periodic expression, in line with other studies of cell cycle genes [64–70]. This method cannot detect non-periodic cell cycle effectors, but our goal here is to model cell cycle based solely on time series gene expression data.

#### The Hopfield model

The Hopfield model [13] is an Ising model whose configuration is defined by *N* spins *σ*_*i*_(*t*) at integer time *t*. The state of each node (gene) takes one of two values, *σ*_*i*_(*t*) = ±1 (over/underexpressed). The coupling matrix *J*_*ij*_ defines the strength and sign of the signal sent from node *j* to node *i*, and its construction is discussed in the following subsection. The total field at node *i* at time *t* is given by

$$\begin{array}{c} h_{i}\left(t\right)=\sum_{j}J_{ij}\sigma_{j}\left(t\right)+h_{i}^{\text{ext}}, \end{array}$$ (1)

where ∑_*j*_ *J*_*ij*_ *σ*_*j*_(*t*) is the internal field at node *i* due to its coupling with all nodes *j* and $h_{i}^{\text{ext}}$ is an optional external field applied to node *i* representing the action of therapeutic compounds, e.g. kinase inhibitors. The dynamical update rule is given by

$$\begin{array}{c} \sigma_{i}\left(t+1\right)=\{\begin{array}{cc} +1 & \text{with}\;\text{probability}\;{\left(1+e^{-2h_{i}\left(t\right)/T}\right)}^{-1}\\ -1 & \text{otherwise} \end{array}, \end{array}$$ (2)

where the factor of 2 in the exponent is conventional and *T* is an effective temperature representing the level of noise (not a physical temperature). Biologically, this noise represents the effects of all kinds of biochemical fluctuations present in cells. Note that for *h*_*i*_(*t*) → ±∞, *σ*_*i*_(*t* + 1) = ±1; for *T* → ∞, *σ*_*i*_(*t* + 1) = ±1 with equal probability; and for *T* → 0, *σ*_*i*_(*t* + 1) = sign (*h*_*i*_(*t*)).

The update rule from Eq 2 may be implemented in various ways. The synchronous scheme updates the state of all nodes in the system at every time step, but this is sensible only if the simulated system has a central pacemaker coordinating the activity of all nodes. A more appropriate choice for decentralized systems like gene regulatory networks is the asynchronous scheme in which the state of a randomly chosen subset of nodes is updated at each time step. Here, we use the asynchronous scheme with update probability 0.2 for each node.

#### Coupling matrix

In the canonical Hopfield model, the coupling matrix is constructed to store a set of *p* linearly independent (i.e. distinct) Boolean patterns $\xi_{i}^{\mu }=\pm 1$ as point attractors, where *i* = 0, 1, …, *N* − 1 is the node index and *μ* = 0, 1, …, *p* − 1 is the pattern index. The point attractor coupling matrix $J_{ij}^{\prime }$ is given by

$$\begin{array}{c} J_{ij}^{\prime }=\frac{1}{Np}\sum_{\mu \nu }\xi_{i}^{\mu }{\left(Q^{-1}\right)}_{\mu \nu }\xi_{j}^{\nu }\;, \end{array}$$ (3)

where [15, 71]

$$\begin{array}{c} Q_{\mu \nu }=\frac{1}{N}\sum_{i}\xi_{i}^{\mu }\xi_{i}^{\nu }\;\text{.} \end{array}$$ (4)

With this coupling matrix and *T* = 0, if at some time *t* the configuration is given by $\sigma_{i}\left(t\right)=\xi_{i}^{\mu }+\delta_{i}$ for a small perturbation *δ*_*i*_, then $\lim_{t\to \infty }\sigma_{i}\left(t\right)=\xi_{i}^{\mu }$. Note that this formulation means $\pm \xi_{i}^{\mu }$ are both attractors of the system.

A simple modification [19] to Eq 3 produces a cyclic attractor coupling matrix ${\tilde{J}}_{ij}$, constructed according to

$$\begin{array}{c} {\tilde{J}}_{ij}=\frac{1}{Np}\sum_{\mu \nu }\xi_{i}^{{\text{mod}}_{p}\left(\mu +1\right)}{\left(Q^{-1}\right)}_{\mu \nu }\xi_{j}^{\nu }\;\text{.} \end{array}$$ (5)

At *T* = 0, this coupling matrix cyclically maps through the sequence of *p* patterns

$$\begin{array}{c} \xi_{i}^{0}\to \xi_{i}^{1}\to \ldots \to \xi_{i}^{p-2}\to \xi_{i}^{p-1}\to \xi_{i}^{0}\to \xi_{i}^{1}\to \ldots \end{array}$$ (6)

or their negatives. For the remainder of this article, all attractor indexing is understood to be modulo *p*.

A delayed cyclic Hopfield model may be constructed by combining the point and cyclic attractor matrices into one coupling matrix,

$$\begin{array}{c} J_{ij}\left(\lambda \right)=\left(1-\lambda \right)J_{ij}^{\prime }+\lambda {\tilde{J}}_{ij}\;, \end{array}$$ (7)

for an adjustable transition strength parameter λ with 0 ≤ λ ≤ 1. If $\sigma \left(t\right)=\xi_{i}^{\mu }$, λ ≪ 1, and *T* = 0, the point attractor term dominates and *σ*_*i*_(*t*) = *σ*(*t* + Δ*t*) for all Δ*t* = 1, 2, …. If *T* > 0, however, stochastic fluctuations eventually push the configuration out of the basin of attraction of the *μ*^th^ attractor and into the (*μ* + 1)^th^ basin, then eventually to the (*μ* + 2)^th^ basin, and so on. The dynamics of the delayed cyclic Hopfield model are thus governed by noise-induced transitions.

Due to the sinusoidal nature of the gene expression in these CC data sets, however, the attractors are structured such that $\xi_{i}^{\mu }=-\xi_{i}^{\mu +4}$, making *Q*_*μν*_ rank deficient and thus noninvertible. Because the definition of *J*_*ij*_ automatically guarantees that if any sequence $\left\{+\xi_{i}^{\mu }\right\}$ is an attractor, then $\left\{-\xi_{i}^{\mu }\right\}$ is also an attractor, encoding the sequence

$$\begin{array}{c} \xi_{i}^{0}\to \xi_{i}^{1}\to \xi_{i}^{2}\to \xi_{i}^{3}\to \xi_{i}^{4}(=-\xi_{i}^{0}) \end{array}$$ (8)

automatically encodes the sequence

$$\begin{array}{c} \xi_{i}^{4}\to \xi_{i}^{5}\to \xi_{i}^{6}\to \xi_{i}^{7}\to \xi_{i}^{0}(=-\xi_{i}^{4})\;\text{.} \end{array}$$ (9)

In this special case of sinusoidal trajectories, the limits of summation in Eqs 3–5 need only run over the first four indices, *μ* = 0, 1, 2, 3.

Finally, to reflect the fact that real gene regulatory networks are sparse, weak edges were removed by setting all elements of the coupling matrix with |*J*_*ij*_| < median(|*J*|) to zero, where |*J*| is element-wise absolute value. The cyclic attractor was not preserved when using stricter thresholds due to the reduced capacity of diluted Hopfield networks [17].

#### Biological interpretation of the dynamics

Extracting biological meaning from this model requires defining some convenient coarse-grained quantities. The overlap of the state vector *σ*_*i*_(*t*) with the *μ*^th^ pattern is given by

$$\begin{array}{c} m^{\mu }\left(t\right)=\frac{1}{N}\sum_{i}\sigma_{i}\left(t\right)\xi_{i}^{\mu }\;, \end{array}$$ (10)

where −1 ≤ *m*^*μ*^(*t*) ≤ + 1. The overlap measures the similarity between the (discretized) experimental and simulated gene expression profiles, and *m*^*μ*^(*t*) = + 1 means there is perfect agreement between the simulated cell’s expression and pattern *μ*.

A single configuration vector *σ*_*i*_(*t*) represents the expression profile of a single cell. For many cells *κ*, let *σ*_*ik*_(*t*) be the expression of gene *i* in cell *k*. Define

$$\begin{array}{c} m_{k}^{\mu }\left(t\right)=\frac{1}{N}\sum_{i}\sigma_{ik}\left(t\right)\xi_{i}^{\mu } \end{array}$$ (11)

as the overlap of cell *k* with attractor *μ*. Because the microarray and RNA-seq data used here report the gene expression averaged over many cells, it is appropriate to define the population-averaged (i.e. ensemble-averaged) expression,

$${\langle \sigma_{i}\left(t\right)\rangle }_{K}=\frac{1}{\kappa }\overset{\kappa -1}{\underset{k=0}{\sum }}\sigma_{ik}\left(t\right)\;,$$ (12)

which has $-1\leq {\langle \sigma_{i}\left(t\right)\rangle }_{K}\leq +1$.

Rather than work with a continuous vector quantity like $m_{k}^{\mu }\left(t\right)$, each cell can simply be identified as being in a discrete phenotypic state at any given time. Define the state of cell *k* as

$$\begin{array}{c} s_{k}\left(t\right)=\underset{\mu }{arg max}m_{k}^{\mu }\left(t\right)\;, \end{array}$$ (13)

i.e. the index of the attractor with maximum overlap, which may be interpreted as cell *k*’s phenotype. To better understand population-level dynamics, define the discrete probability distribution *P*_*μ*_(*t*) as the fraction of *κ* cells with *s*_*k*_(*t*) = *μ*; that is, *P*_*μ*_(*t*) is the probability that a randomly chosen cell is in state *μ* at time *t*. Finally, define the time-averaged distribution of states as

$${\langle P_{\mu }\rangle }_{T}=\frac{1}{\tau }\overset{\tau -1}{\underset{t=0}{\sum }}P_{\mu }\left(t\right)$$ (14)

for a window of time *τ*.

For each data set, *T*, λ, and the single-node update probability were tuned to the “edge of chaos” [72] such that the cyclic attractor was preserved and the time between transitions was approximately constant, but the system was sensitive enough to perturbations that some targeted inhibitions produced noticeable changes in ${\langle P_{\mu }\rangle }_{T}$. See S1 Table for a list of parameters used for each data set.

#### Gene inhibition optimization

In this application, the goal is to identify perturbations that halt or retard the encoded cyclic attractor. A standard genetic algorithm (GA; explained in S1 Text) was employed to identify an optimal control field $h_{i}^{\text{opt}}$ that maximized a given objective function $f(h_{i}^{\text{ext}})$,

$$\begin{array}{c} h_{i}^{\text{opt}}=\underset{h_{i}^{\text{ext}}}{arg max}f(h_{i}^{\text{ext}})\;, \end{array}$$ (15)

where $h_{i}^{\text{ext}}$ is the control vector given by

$$\begin{array}{c} h_{i}^{\text{ext}}=\{\begin{array}{cc} -\infty & \text{if}\;\text{gene}\;i\;\text{is}\;\text{targeted}\\ 0 & \text{otherwise} \end{array} \end{array}$$ (16)

for a fixed number of targets (nonzero elements) *n*_targ_. Only negative control fields are used here to simulate the effects of targeted gene inhibition from pharmaceutical compounds. The objective function used here is ${\langle P_{\mu }\rangle }_{T}$, meaning that the optimal control field maximizes the time-averaged number of cells occupying a particular attractor state *μ*. This search was conducted across all attractors *μ* to determine the controllability of each attractor state. Note that while the numerical value of ${\langle P_{\mu }\rangle }_{T}$ depends on the choice of parameters such as *T*, λ, and the single-node update probability, varying these parameters by ±5% gave comparable results for the optimal sets of controllers.

### Dynamical behavior

**Fig 2:**
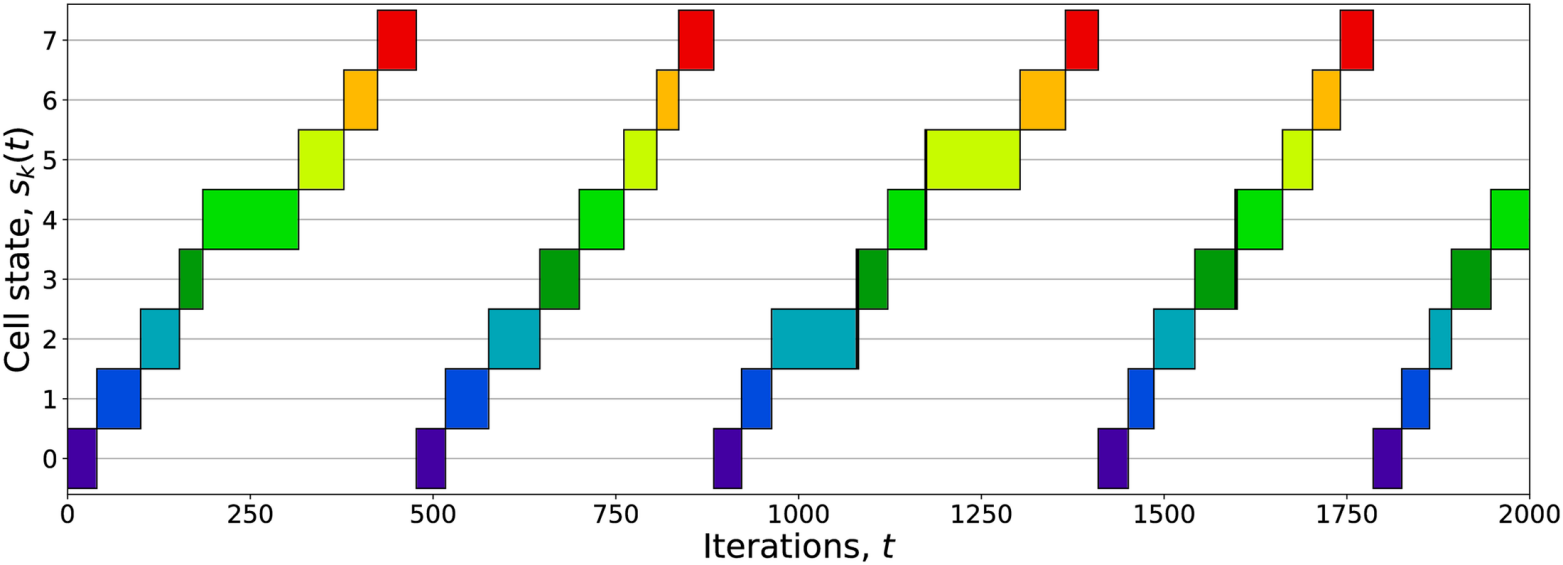
Unperturbed cell state versus time. Boxes indicate *s*_*k*_(*t*), i.e. the index of the attractor with maximum overlap at time *t*. The system began with the configuration $\sigma_{i}\left(0\right)=\xi_{i}^{0}$ and was allowed to evolve according to the Hopfield signaling rules with zero external field, mapping cyclically through the set of eight attractors. The pattern and cycle durations vary due to the system’s stochasticity.

Fig 2 shows the time evolution of *s*_*k*_(*t*) for a single simulated cell using the attractors derived from [64]. As expected, the system progresses cyclically through the eight attractor states. The duration of each cycle varies somewhat due to the stochasticity in the update rule from Eq 2.

**Fig 3:**
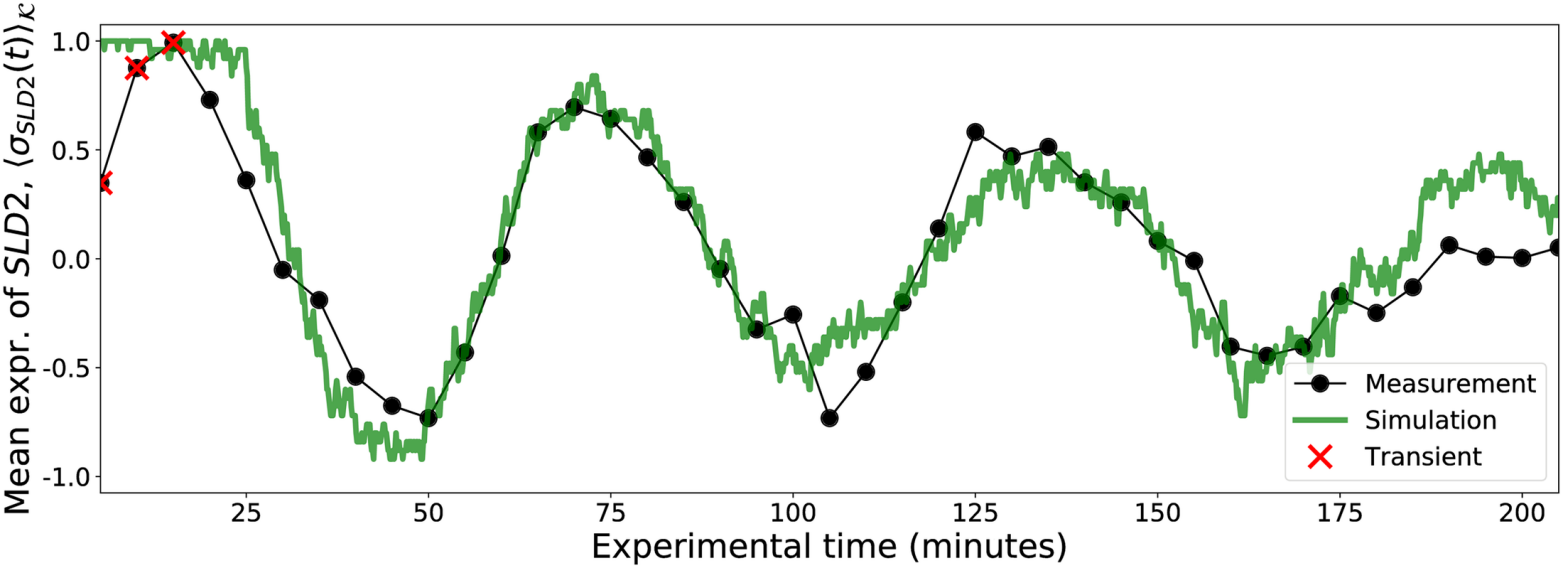
Measured and simulated gene expression. The measured expression of the *S. cerevisiae* gene *SLD2* from [64] was scaled to the range [−1, +1] and is shown as a black beaded curve, and the population-averaged expression of the same gene as defined in Eq 12 for *κ* = 50 cells is shown in green, with the *t* axis rescaled by eye to match experimental time. Transient points (red X’s) were ignored when fitting Eq 17.

Although the gene expression for each simulated cell *k* has *σ*_*ik*_(*t*) = ±1, the population-averaged expression has $-1\leq {\langle \sigma_{i}\left(t\right)\rangle }_{K}\leq +1$, and for many cells initially synchronized with $\sigma_{ik}\left(0\right)=\xi_{i}^{0}$ for all *k*, ${\langle \sigma_{i}\left(t\right)\rangle }_{K}$ successfully recovers the experimentally observed decaying sinusoidal gene expression. Fig 3 shows a comparison between the experimental expression *x*_*i*_(*t*) from the Eser data set and the mean simulated expression ${\langle \sigma_{i}\left(t\right)\rangle }_{K}$ with *κ* = 50 for *i* = *SLD2*, one of the genes responsible for initiating DNA replication in *S. cerevisiae* [73, 74]. The simulation time *t* was rescaled by eye to align the simulated and experimental curves (so that 1 minute corresponds to approximately 7.5 iterations).

**Fig 4:**
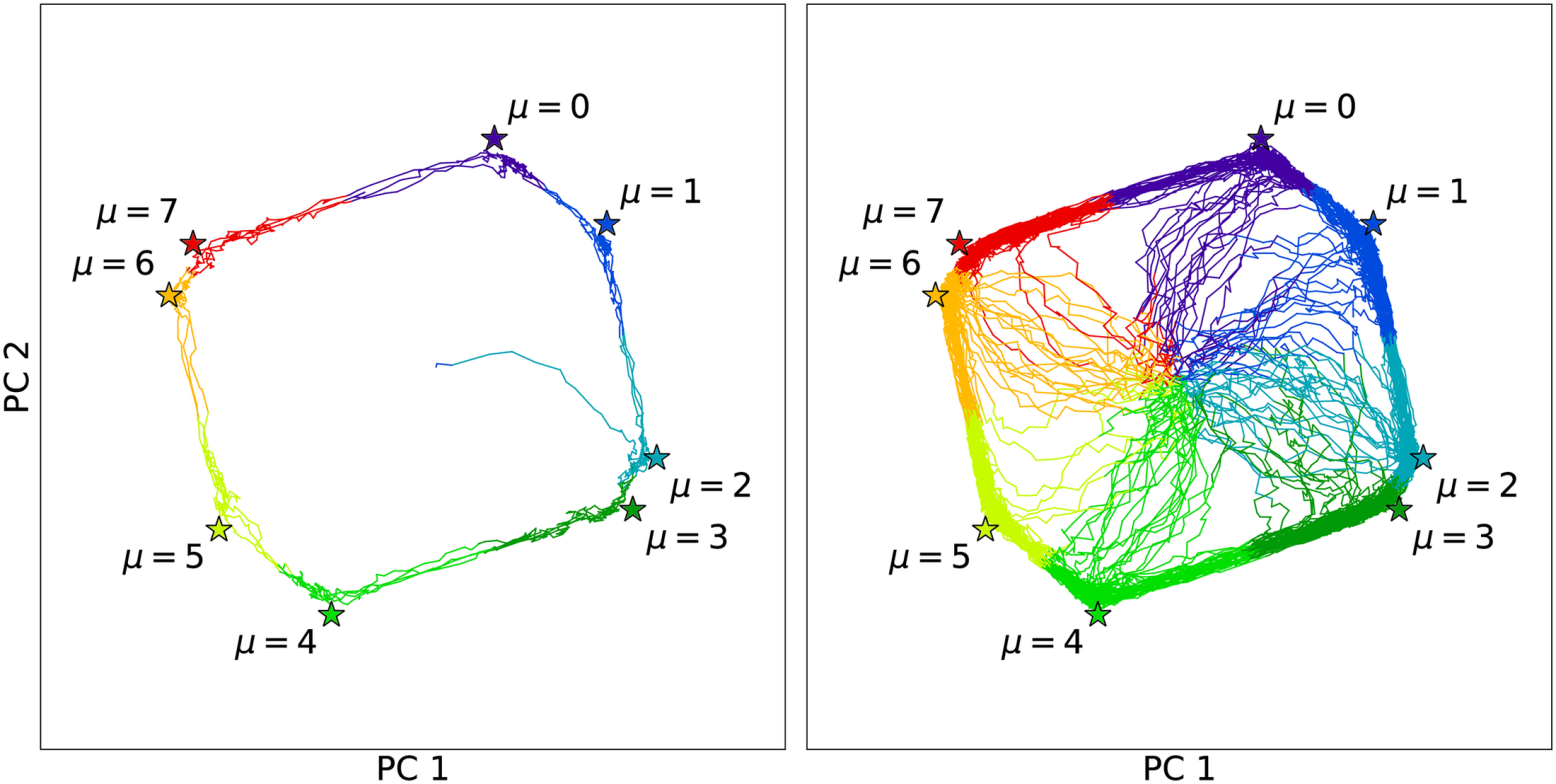
Principal component projection of unperturbed cell trajectories. The simulated single cell (left panel) and 100 cells (right panel) began with random initial states (projected near the center of the plot), but quickly settled into the encoded cycle. Line segments were colored according to *s*_*k*_(*t*), i.e. which of the eight attractors (labeled stars) had maximum overlap at time *t*.

Trajectories can be visualized by projecting them onto the first two principal components (PCs) of the attractor configurations. Fig 4 shows the eight attractors as stars, and a single cell trajectory (left panel) and 100 cell trajectories (right panel) with random initial states as curves with line segments colored according to *s*_*k*_(*t*) (as computed in the full *N*-dimensional space). Although the cells begin nearly equidistant from all $\xi_{i}^{\mu }$, they quickly relax into encoded cycle. S1 Video shows an animation of a system of *κ* = 50 cells with random initial conditions projected onto the same PCs, where cells (circles) are colored according to *s*_*k*_(*t*). As with the cells shown in Fig 4, all initially random configurations eventually converge to the cycle. S2 Video shows an animation of *κ* = 50 cell trajectories with $\sigma_{ik}\left(0\right)=\xi_{i}^{0}$. As time progresses, the phases of the initially synchronized cells slowly decohere because cells stochastically and independently transition between attractors due to the finite temperature in Eq 2.

S3 Video demonstrates the effect of temperature on the dynamics. 50 cells were given random initial states, and the temperature was increased and decreased in steps. Cells rarely escape the eight attractor states for *T* = 0.045, and one cell becomes stuck near the center in a spurious attractor (unintentional metastable states that arise from the model’s nonlinearity). At *T* = 0.06, fluctuations allow the cells to transition somewhat regularly through the encoded cycle, and the cell trapped in the spurious attractor eventually escapes and joins the cycle. At *T* = 0.09 the cells begin to noticeably diverge from the eight attractor states, but still collectively display a net clockwise flow. The noise is too great for the cells to follow the cycle for *T* = 0.15, but lowering the temperature again returns cells to the cycle. This illustrates the fact that the cycle is preserved only for intermediate temperatures: cells become “frozen” in intended or spurious attractor states at low temperatures, but at high temperatures the noise is too great and the couplings between genes become irrelevant to the dynamics.

### Optimal control fields

**Fig 5:**
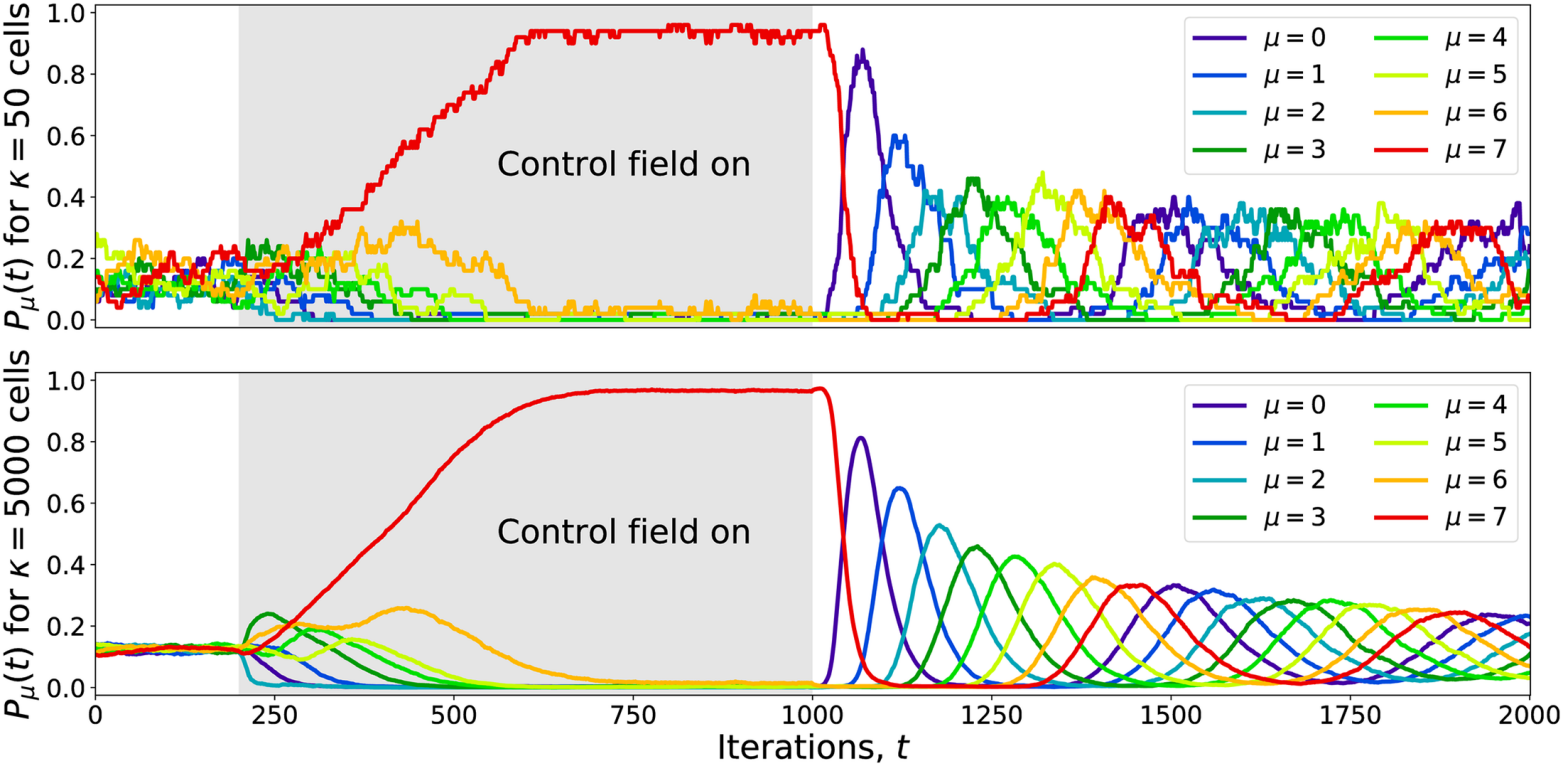
Cell state synchronization by targeted inhibition for 50 and 5000 cells. Cells were initialized with random states at *t* = −200 (not shown) and allowed to relax into the cyclic attractor so that *P*_*μ*_(0 ≤ *t* < 200) ≈ 1/8. A set of eight genes was inhibited with an external control field over the range 200 ≤ *t* < 1000, fixing most cells near the *μ* = 7 state. After removing the control field, the cells resumed moving through the cycle with initially synchronized phases that slowly broaden. Eventually the system returns to a desynchronized state, *P*_*μ*_(*t* → ∞) ≈ 1/8.

The GA was used to identify some effective combinations of gene targets that slowed progress through the cyclic attractor for varying numbers of targets, *n*_targ_. The GA found, for example, that inhibiting the set of eight *S. cerevisiae* genes *HEK2*, *PRR1*, *QRI1*, *RFC4*, *STB1*, *TDA7*, *VPS17*, and *ZIM17* was sufficient to trap ∼95% of cells in the *μ* = 7 state. The effects of this control field on the time evolution of *P*_*μ*_(*t*) for *κ* = 50 and *κ* = 5000 are shown in Fig 5. Cells were given random, independent initial states at *t* = −200 (not shown), quickly settling into the cyclic attractor with evenly distributed phases so that *P*_*μ*_(0 ≤ *t* < 200) ≈ 1/8. The control field was activated at *t* = 200, causing the cells to accumulate in the *μ* = 7 state. The field was then disabled at *t* = 1000, allowing the cells to resume cycling with initially synchronized phases, as shown by the sequence of oscillations in *P*_*μ*_(*t* > 1000). The stochastic nature of the transitions causes the cells’ phases to slowly spread so that *P*_*μ*_(*t* → ∞) ≈ 1/8, eventually returning the system to a desynchronized state.

**Fig 6:**
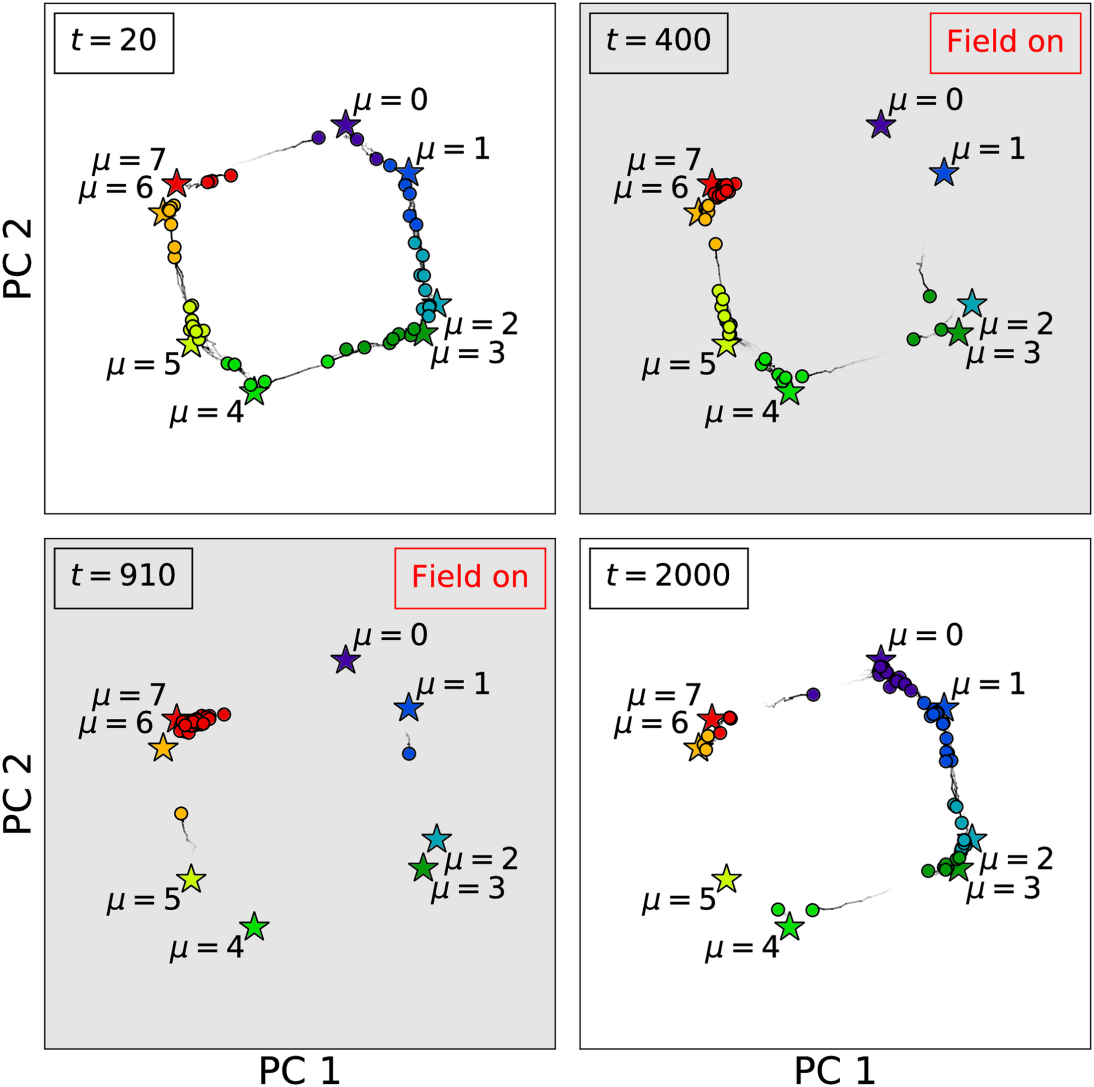
Principal component projection of 50 cell trajectories. The trajectories used to make the *κ* = 50 panel of Fig 5 were projected onto the the first two principal components (PCs) of the attractor array $\xi_{i}^{\mu }$ (labeled stars). Cells (circles) are colored according to the closest attractor as computed by Eq 13. When the external field is activated, most cells become trapped in the *μ* = 7 state, although occasionally cells break from the group and complete another circuit before becoming trapped again. After the external field is removed, the cells eventually return to a desynchronized state. See S4 Video for an animation of these trajectories.

The effects of this control field can also be visualized using a PC projection as shown in Fig 6 and S4 Video. The same set of *κ* = 50 trajectories from Fig 5 was projected onto the attractors’ PCs, with cells colored according to *s*_*k*_(*t*). The control field manages to fix most cells near the *μ* = 7 state, but as shown in the *t* = 910 panel in Fig 6, fluctuations occasionally push individual cells out of the *μ* = 7 basin and back into the cycle.

Further searches were constrained to inhibiting between 1 and 38 kinases in HeLa cells and between 1 and 12 kinases in *S. cerevisiae*. For nearly all numbers of targets, the GA found *μ* = 2 (M phase) to be the most controllable attractor in HeLa cells and *μ* = 7 (S phase) in *S. cerevisiae*, as shown in S1 and S2 Figs, respectively. In HeLa cells, for example, an optimal score of ${\langle P_{2}\rangle }_{T}\approx 0.230$ was obtained for *n*_targ_ = 4 (0.55% of the 722 periodic genes) when inhibiting the genes *AURKB*, *NEK1*, *TTK* (all involved in the formation and operation of the spindle apparatus), and *WEE1* (a G2/M checkpoint regulator involved with DNA damage repair). The effects of inhibiting *AURKB* and *TTK* are well documented. *AURKB* is targetable by the kinase inhibitor barasertib, found to be effective in combating small-cell lung cancer *in vitro* and in xenografts [75]. *TTK* (also known as *MPS1*) is targetable by the kinase inhibitors Mps1-IN-1 and AZ3146. Mps1-IN-1 has been found to be an effective tool to inhibit proliferation in an array of cancer cell lines, including HeLa cells [76]. AZ3146 has been effective against hepatocellular carcinoma (liver cancer) *in vitro* [77] and pancreatic ductal adenocarcinoma *in vitro* [78]. siRNAs and shRNAs have also been used to inhibit TTK in pancreatic ductal adenocarcinoma [78].

## Discussion

Above we presented a delayed cyclic Hopfield model designed to store CC time series gene expression data from synchronized *S. cerevisiae* and HeLa cells, and the behaviors of both individual cells and populations of cells were studied. The dynamics of populations of cells successfully recreated the experimental gene expression data, including the slow decoherence of initially synchronized cells due to the stochastic transitions between attractors. Optimal control fields that freeze or stall the cyclic attractor by inhibiting only a small number of genes were identified. These predictions could be experimentally tested using kinase inhibitors, micro RNAs, short interfering RNAs, or knockout studies and confirmed simply by counting the number of cells as a function of time compared to a control with no inhibitors.

Admittedly, there are several limitations to this model. Additionally, although using the temporal ordering of the time series gene expression samples provides more information about potentially causal relationships than static samples, the Hopfield model is ultimately an effective model that builds gene-gene couplings from pairwise correlations in gene expression, thereby capturing direct, indirect, and spurious relationships between genes. Independently derived network information with experimentally confirmed molecular regulatory interactions could perhaps be used to refine the construction of the coupling matrix. Other data sets that explore a more diverse set of expression states other than a simple cycle could also reduce the number of spurious couplings. We are currently developing these kinds of non-cyclic models and time series data sets in a separate project.

Our approach can be generalized and improved in many ways. This incarnation of the model causes simulated cells to continuously undergo CC with no G0 (resting) phase. Adding a relatively stable G0 attractor between the M and G1 phases could cause cells to pause between cycles. A GA search could then be conducted to find the best sets of inhibition targets to freeze cells in the G0 state, or to find the best sets of targets to stimulate entry into CC, mimicking the effects of environmental signals such as growth factors.

We chose to discretize the continuous gene expression data using a traditional two-state model, which assumes that each gene is either fully activated or fully deactivated. Using a multi-level Hopfield model [79] could better reflect the continuous nature of gene expression data and potentially improve the search results. This model can also incorporate additional omics information, e.g. proteomics and metabolomics, simply by increasing the number of nodes in the system. We plan to explore this option as more multi-omics time series data sets become available. Single-cell experimental techniques and analytical tools are also rapidly improving in quality, decreasing in cost, and gaining in popularity [80–82], and using techniques like pseudo-temporal ordering [83] could allow the Hopfield model to encode single-cell RNA-seq data as well.

Although the above simulated populations of cells exhibit intriguing dynamical and statistical properties, they behave as completely homogeneous, non-interacting particles. The importance of cell-cell communication and interactions in populations of cells has been demonstrated in a variety of systems including bacterial quorum sensing [84] and community spatial patterning [85], neuron synchronization in circadian rhythm [86], and various forms of cancer [87–91]. As with many nonlinear systems, even seemingly minor changes can produce dramatically different outcomes. More complex extensions to our model could incorporate cell-cell communication by, for example, adding couplings between known signaling molecules and receptors between different cells, and could even allow for interactions between heterogeneous cell types. This would increase the computational complexity of the model, but could better reflect the underlying biology.

## Methods

### Gene expression fitting

In order to encode these CC data sets into the Hopfield model, periodic genes needed to be identified, their frequencies and phases computed, and their expression converted from continuous to Boolean form. SciPy’s Trust Region Reflective method [92] was used to identify genes *i* with periodic expression *x*_*i*_(*t*) by fitting to the form

$$\begin{array}{c} x_{i}\left(t\right)=a_{i}e^{-b_{i}t}\cos(\omega_{i}t-\phi_{i})+x_{i0} \end{array}$$ (17)

for amplitude *a*_*i*_, decay rate *b*_*i*_, angular frequency *ω*_*i*_, phase *ϕ*_*i*_, and asymptotic mean expression *x*_*i*0_. The first several time points were ignored to avoid fitting the parameters of Eq 17 to chemically perturbed (transient) states. A gene was labeled periodic if the maximum relative uncertainty in its parameters from the fit,

$$\begin{array}{c} r_{i}^{\text{max}}=\max\{\frac{\delta x_{i0}}{x_{i0}},\frac{\delta a_{i}}{a_{i}},\frac{\delta b_{i}}{b_{i}},\frac{\delta \omega_{i}}{\omega_{i}},\frac{\delta \phi_{i}}{2\pi }\}\;, \end{array}$$ (18)

was less than the thresholds defined in S1 Table. (Note that because the HeLa data covers only a little more than one period, many of the *b*_*i*_’s were approximately zero. This resulted in extremely large $r_{i}^{\text{max}}$ despite the remaining variables having small uncertainty. The *δb*_*i*_/*b*_*i*_ term was thus ignored for the HeLa data.) Once all frequencies {*ω*_*i*_} for periodic genes were computed, the frequency was fixed to the mean frequency $\bar{\omega }$ and the fits were recomputed for each periodic gene using the form

$$\begin{array}{c} x_{i}\left(t\right)=a_{i}e^{-b_{i}t}\cos(\bar{\omega }t-\phi_{i})+x_{i0}\;, \end{array}$$ (19)

thus producing the final set of continuous phases {*ϕ*_*i*_}. Fig 1A shows a heat map of the expression of all periodic genes detected in the Eser data set sorted by their fitted phases, and Fig 1B shows the same genes with the fitted expression curves. These fitted curves were converted from continuous values *x*_*i*_(*t*) ≥ 0 to Boolean values *ξ*_*i*_(*t*) = ±1 (over/underexpressed) by assigning

$$\begin{array}{c} \xi_{i}\left(t\right)=\mathrm{sign}\left(x_{i}\left(t\right)-x_{i0}\right) \end{array}$$ (20)

as shown in Fig 1C. Finally, one CC period was divided into eight uniformly spaced states $\left\{\xi_{i}^{\mu }\right\}=\left\{\xi_{i}^{0},\xi_{i}^{1},\ldots ,\xi_{i}^{7}\right\}$. These states, shown in Fig 1D, were used as attractors in the Hopfield model.

### Determining cell cycle phase

The approximate CC phase for each attractor *μ* in the HeLa data set was determined using a set of marker genes [65, 93] and over-representation analysis using a hypergeometric distribution to calculate *p*-values with the Benjamini-Hochberg procedure [94] to correct for multiple hypothesis testing. All genes *i* with $\xi_{i}^{\mu -1}=-1$ and $\xi_{i}^{\mu }=+1$ were used as the *μ*^th^ input set, and all detected cyclic genes were used as the background. *μ* = 2 showed the activation of the G2/M checkpoint marker *CCNA2* and was enriched for the gene ontology (GO) term cell division (*p* = 3.1 × 10^−2^). *μ* = 3 showed the activation of the G2, G2/M, and M markers *BUB1*, *CCNB1*, *NEK2*, and *PLK1*, and was enriched for signaling by Rho GTPases, cell division, midbody, mitotic prometaphase, mitotic nuclear division, and small GTPase mediated signal transduction (*p* = 3.7 × 10^−3^); M phase (*p* = 6.2 × 10^−3^); resolution of sister chromatid cohesion (*p* = 7.6 × 10^−3^); mitotic anaphase, spindle pole, and RHO GTPase effectors (*p* = 3.0 × 10^−2^); and mitotic metaphase and anaphase (*p* = 3.8 × 10^−2^). *μ* = 6 expressed the late G1, G1/S, and S markers *CCNE1*, *CDC6*, *E2F1*, *MCM2*, and *SLBP*, and was enriched for DNA replication initiation (*p* = 5.9 × 10^−4^); G1/S-specific transcription, G1/S transition, DNA replication pre-initiation, M/G1 transition, and assembly of the pre-replicative complex (*p* = 4.3 × 10^−2^); and G1/S transition of mitotic cell cycle (*p* = 4.4 × 10^−2^). *μ* = 7 showed the activation of the G1/S and S markers *CDK2* and *CDKN2AIP*. The database yeastgenome.org [95] was used to determine the CC phases for the Eser *S. cerevisiae* data set. *μ* = 0 was enriched for the GO term DNA replication (*p* = 2.02 × 10^−12^), indicating an attractor in the S phase of CC. *μ* = 2 was enriched for mitotic spindle organization (*p* = 2.3 × 10^−3^) indicating the beginning of mitosis in *S. cerevisiae*. *μ* = 6 from Eser was enriched for the GO term pre-replicative complex assembly involved in nuclear cell cycle DNA replication (*p* = 3.0 × 10^−5^), indicating an attractor at the end of G1 phase as the cells prepare for DNA replication.

## Supporting information

S1 Video50 cell trajectories with random initial conditions.Data was projected onto the first two principle components of the attractor array $\xi_{i}^{\mu }$. Attractors are shown as stars, and cells are shown as circles. Cell colors are assigned using *s*_*k*_(*t*) as measured in the full *N*-dimensional space. All cells *k* were given random initial conditions *σ*_*ik*_ = ±1 with equal probability for all *i* and *k*, but eventually converge to the cyclic attractor.(AVI)

S2 Video50 cell trajectories with identical initial conditions.See the caption of S1 Video for an explanation of the projection and colors. All cells *k* were initially synchronized with $\sigma_{ik}\left(t\right)=\xi_{i}^{0}$, but progress through the cycle stochastically, causing the distribution of *s*_*k*_(*t*) to broaden.(AVI)

S3 Video Effects of temperature on 50 cell trajectories. See the caption of S1 Video for an explanation of the projection and colors. All cells were given initial random states, and the temperature was increased and decreased in steps as shown in the top panel.
(AVI)

S4 Video Principal component projection of 50 cells being synchronized. See the caption of S1 Video for an explanation of the projection and colors. This video is an animation of the trajectories used in Figs 5 and 6.
(AVI)

S1 Text Explanation of genetic algorithm. (PDF)

S1 Table List of parameters used for each data set. (XLSX)

S2 Table Enrichment results for each attractor from the Eser data set. (XLSX)

S3 Table Enrichment results for each attractor from the Dominguez data set. (XLSX)

S1 Fig GA’s optimal control results versus the number of inhibited kinases for HeLa cells. Each point indicates the best measured score (mean fraction of cells) for the given attractor and number of targets. *μ* = 2 (M phase) is generally the most controllable attractor in HeLa cells.
(PNG)

S2 Fig GA’s optimal control results versus the number of inhibited kinases for *S. cerevisiae*. Each point indicates the best measured score (mean fraction of cells) for the given attractor and number of targets. *μ* = 7 (S phase) is generally the most controllable attractor in *S. cerevisiae*.
(PNG)

S1 File Raw yeast time series gene expression data. Taken from [64].
(CSV)

S2 File Raw HeLa time series gene expression data. Taken from [65].
(XLSX)
