# Supplementary material for: Cell cycle time series gene expression data encoded as cyclic attractors in Hopfield systems
Source: PLoS Comput Biol. 2017 Nov 17;13(11):e1005849. doi: 10.1371/journal.pcbi.1005849 (PMC5711035; doi:10.1371/journal.pcbi.1005849)
Supplement: S1 Text — (PDF) [file pcbi.1005849.s005.pdf]

S1 File: Genetic Algorithm

Quotes are used below to avoid confusion between real, biological genes/genomes and the abstract "genes"/"genomes" used in genetic algorithms. The algorithm used in this paper, shown diagrammatically below, implemented the following basic steps.

1. η agents were constructed by randomly choosing g "genes" out of the pool of G "genes" without replacement.
2. The objective function was evaluated for each agent's "genome."
3. Agents were ranked from best to worst (lowest to highest objective function evaluation), and the bottom x (measured in percent) of agents was removed.
4. Removed agents were replaced by the children of randomly paired parent agents from the top $100\% - x$ of agents. New agents' "genes" were selected from the parents' combined "genomes" without replacement, where the probability for selecting each "gene" was proportional to its number of occurances in the parents' combined "genomes" (either 1 or 2).
5. Each "gene" in both old and new agents had a fixed probability q of mutating to any of the $G - g$ "genes" not already in its "genome." The algorithm then looped back to Step 2 until the minimum objective function evaluation no longer improved between subsequent generations.

Figure 1 shows one example iteration of steps 1-5 for $\eta = 3$, $G = 6$, $g = 3$, and $x = 33\%$.

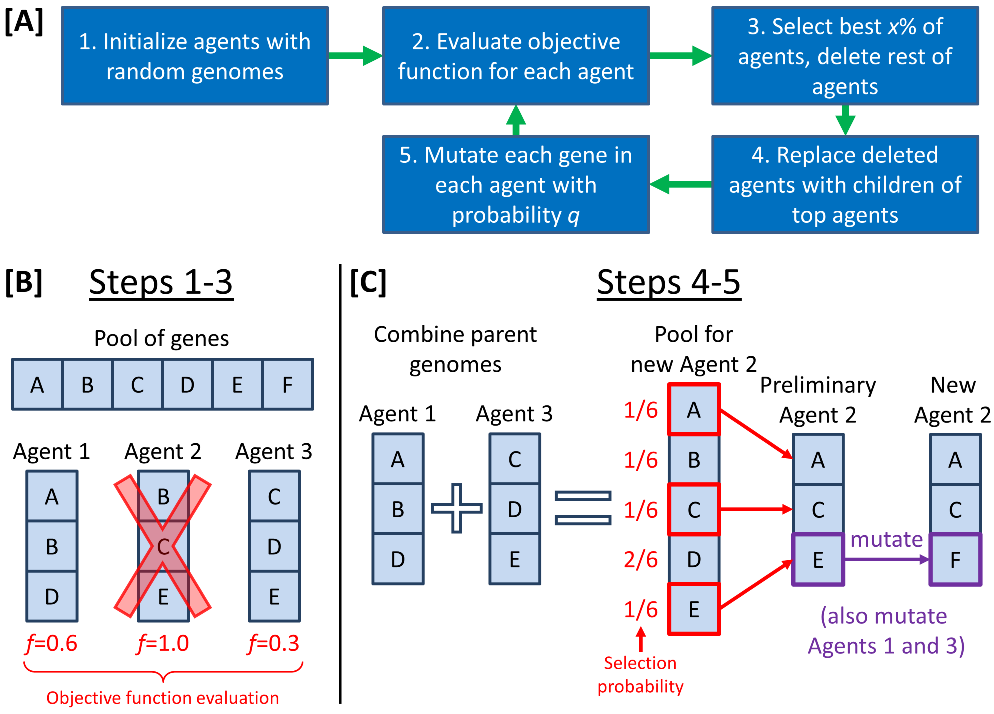

Figure 1: **Genetic algorithm diagram.** Standard genetic algorithm for categorical inputs. A: Algorithm flow chart. B: Example of one iteration of the algorithm for steps 1-3. C: Example of one iteration of the algorithm for steps 4 and 5.
