# Supplementary figures and images for: Cell cycle time series gene expression data encoded as cyclic attractors in Hopfield systems
Source: PLoS Comput Biol. 2017 Nov 17;13(11):e1005849. doi: 10.1371/journal.pcbi.1005849 (PMC5711035; doi:10.1371/journal.pcbi.1005849)

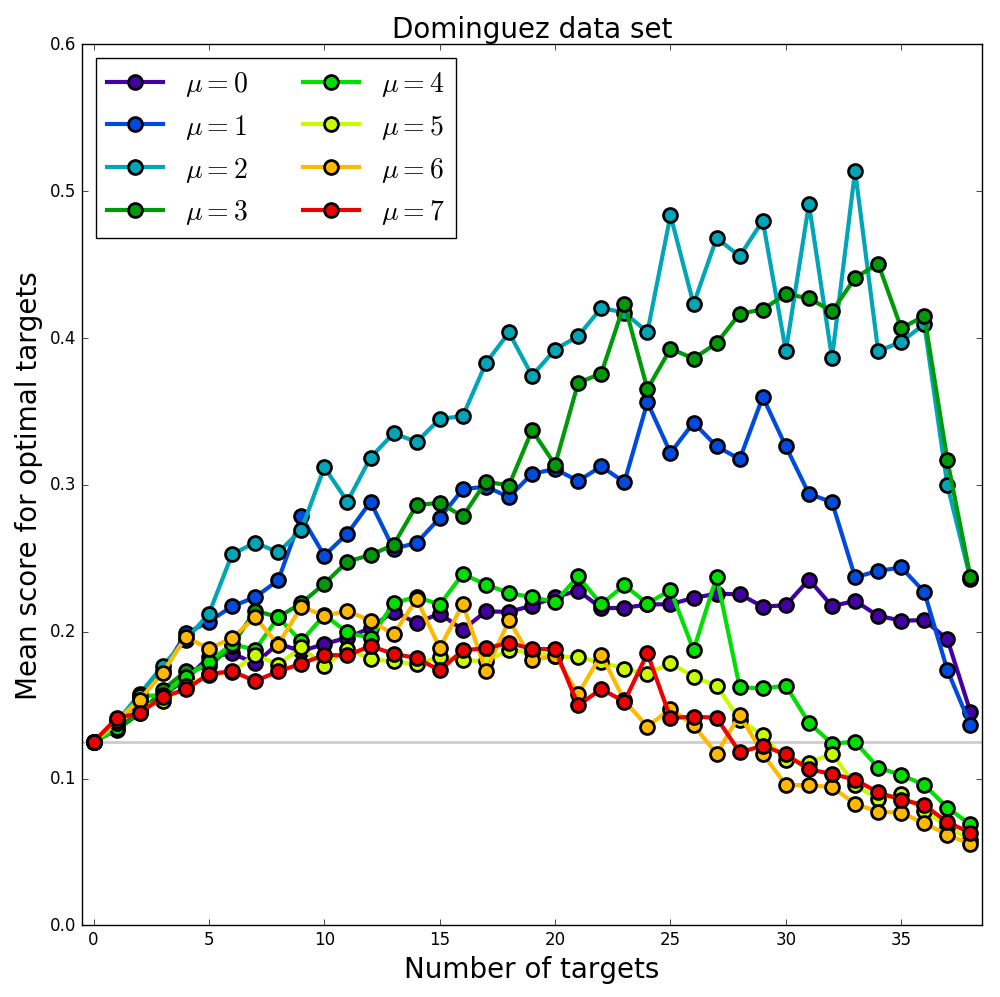

Supplement: S1 Fig — Each point indicates the best measured score (mean fraction of cells) for the given attractor and number of targets. μ = 2 (M phase) is generally the most controllable attractor in HeLa cells. (PNG) [file pcbi.1005849.s009.png]

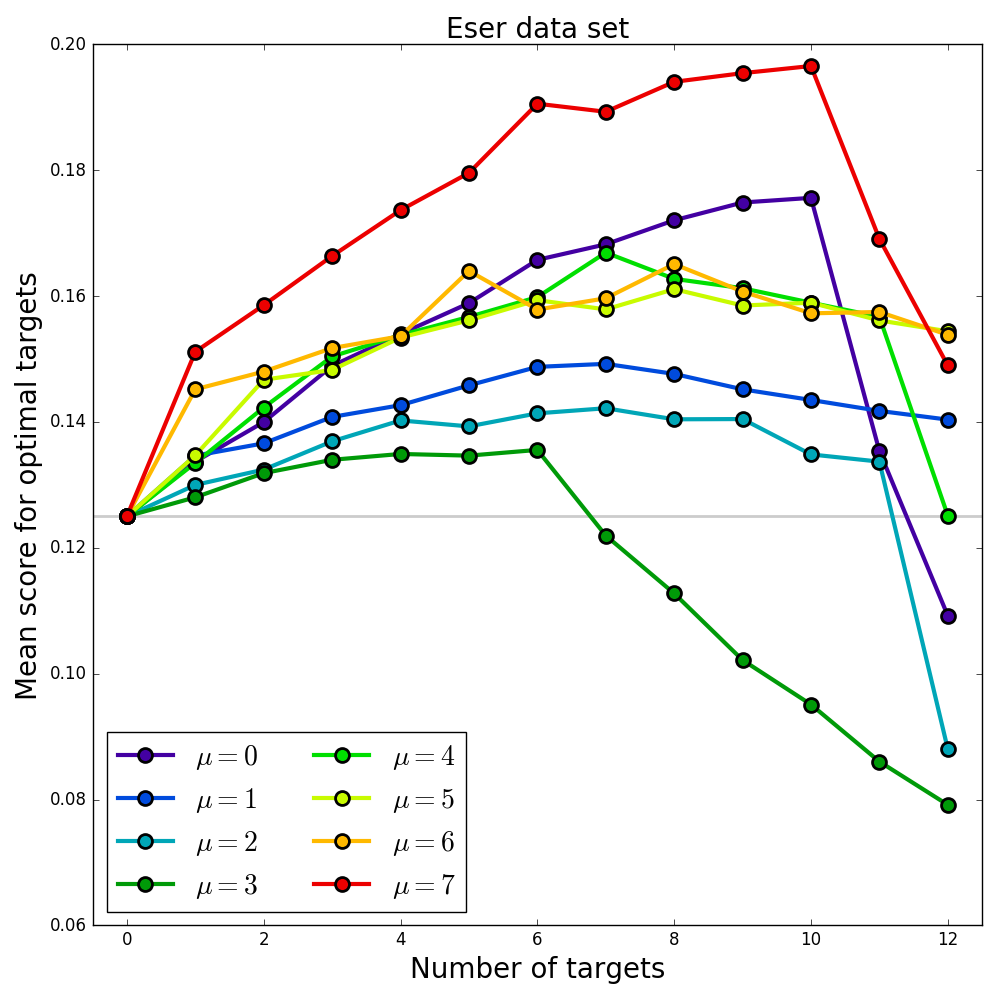

Supplement: S2 Fig — Each point indicates the best measured score (mean fraction of cells) for the given attractor and number of targets. μ = 7 (S phase) is generally the most controllable attractor in S. cerevisiae. (PNG) [file pcbi.1005849.s010.png]
